# Treatment of MSCs with Wnt1a-conditioned medium activates DP cells and promotes hair follicle regrowth

**DOI:** 10.1038/srep05432

**Published:** 2014-06-25

**Authors:** Liang Dong, Haojie Hao, Lei Xia, Jiejie Liu, Dongdong Ti, Chuan Tong, Qian Hou, Qingwang Han, Yali Zhao, Huiling Liu, Xiaobing Fu, Weidong Han

**Affiliations:** 1 Institute of Basic Medicine Science, College of Life Science, Chinese PLA General Hospital, Beijing 100853, China; 2 Department of Medical Administration,Chinese PLA General Hospital, Beijing 100853, China; 3 Central laboratory, Hainan branch of Chinese PLA General Hospital, Sanya, 572013, China; 4 These authors contributed equally to this work.

## Abstract

Hair loss (alopecia) is a common problem for people. The dermal papilla is the key signaling center that regulates hair growth and it engage in crosstalk with the microenvironment, including Wnt signaling and stem cells. In this study, we explored the effects of bone marrow mesenchymal stem cell overexpression of Wnt1a on mouse hair follicle regeneration. Wnt-CM accelerated hair follicle progression from telogen to anagen and enhanced the ALP expression in the DP area. Moreover, the hair induction-related genes were upregulated, as demonstrated by qRT-PCR. Wnt-CM treatment restored and increased DP cell expression of genes downregulated by dihydrotestosterone treatment, as demonstrated by qRT-PCR assays. Our study reveals that BM-MSC-generated Wnt1a promotes the DP's ability to induce hair cycling and regeneration.

Hair loss (alopecia) is a common and distressing problem that can result from a complex range of disorders, including genetic, hormonal, traumatic and iatrogenic events. In particular, the occurrence of androgenetic alopecia (AGA) has increased to comprise 71% of all alopecia cases^1^. Currently, there are several treatment options for alopecia patients, such as wearing a wig, using oral or topical medicines, or surgical management. Drug treatment provides only temporary relief, and the discontinuation of medication may result in immediate depilation. Autologous single follicle and follicular unit transplantation is a reliable surgical option, but the number of donor follicles is limited. Therefore, alternative strategies are urgently needed for hair loss treatment.

The hair follicle (HF) is a regenerating system that undergoes a cyclic process of growth, regression and resting phases (anagen, catagen, and telogen, respectively), and it is composed of both dermal and epidermal compartments^2^. The dermal papilla (DP) is the major dermal compartment and is widely recognized as the key signaling center responsible for maintaining hair growth and controlling hair follicle cycling throughout mammals life^3^,^4^. The microenvironment influences the ability of the DP to induce growth, and a worsened microenvironment can induce hair loss^5^. A previous study demonstrated that the antiapoptotic B-cell lymphoma-2 (Bcl-2) protein is downregulated in human DP cells after cisplatin treatment, leading to DP cell apoptosis and massive hair loss. The androgen activator dihydrotestosterone (DHT), induced increased dickkopf 1 (Dkk-1) overexpression and decreased lymphoid enhancer factor-l (Lef-1) expression in cultured DP cells, which can cause apoptosis of follicular keratinocytes in co-cultured systems^6^,^7^. These results are similar to the phenotype observed in AGA patients, where the hair follicle cycle is gradually interrupted during telogen phase, and terminal scalp hairs are gradually replaced by smaller hairs^8^. Thus, we hypothesize that restoring the DP's ability to induce hair growth well be an effective treatment strategy for alopecia.

Wingless-type mouse mammary tumor virus integration site (Wnt) signaling is a crucial signaling pathway that regulates embryonic and adult hair morphogenesis and regeneration^9^. Studies have previously demonstrated that hair follicle neogenesis can be induced in a 1-cm^2^ full or greater thickness wounds in mice, and when Wnt7a is overexpressed, the number of hair follicles in the wound increases^10^,^11^. The use of Wnt signaling in hair neogenesis is groundbreaking. In addition, Wnt3a expression can maintain anagen gene expression in DP cells and mediate hair induction ability in human hair follicles organ culture^10^. Adenovirus-mediated Wnt10b overexpression regulates the biological switch of hair follicles from telogen to anagen phase induces hair follicle regeneration in mice^12^. However, during hair follicle formation, Wnt1a is first expressed in the epithelial placode, which invaginates into the underlying dermis and joins the dermal condensation germ is the initial signal that stimulates hair follicle formation^13^,^14^. Whether Wnt1a can trigger the hair cycle and regeneration is unclear.

The dermal papilla (DP) is embedded in the hair bulb at the base of the follicle and is surrounded by a variety of stem cells. DP cells induce the hair cycle and regeneration, which requires crosstalk with the stem cell populations^15^. Adipose stem cells (ASCs) that come from the fat layer of mouse skin and can produce platelet-derived growth factor (PDGF), which activates DP cells to promote hair growth^16^. When conditioned media from human amniotic fluid-derived MSCs (AF-MSC-CM) was subcutaneously injected around full thickness wounds in rats, wound healing was accelerated, but more surprisingly, hair regrew at the wound site^17^. There are some reports suggesting that bone marrow mesenchymal stem cells (BM-MSCs) can also be used in animal models wound repair and can enhance hair regrowth^18^. MSCs enhance wound healing and promote hair follicle regeneration through the differentiation or release of growth factors when injected in the wound bed in excisional wound splinting models^19^. However, it is unclear whether MSCs interact with DP cells to triggers hair regeneration.

In our study, we prepared conditioned media (CM) from the supernatant of cultured BM-MSCs Wnt1a-overexpressing (Wnt-CM) and investigated its effect on mouse hair regeneration. Our results revealed that Wnt-CM accelerate the biological progression of hair follicles from the telogen to anagen phase and increases the number of hairs. *In vitro* experiments demonstrated that Wnt-CM can restores and maintains the hair induction ability of intermediate follicles DP cells damaged by DHT. These results indicate that Wnt1a overexpression by BM-MSCs can promote hair cell cycle progression and induce hair regeneration by actively facilitating the induction ability of mouse DP cells in mice.

## Results

### Wnt1a overexpression in BM-MSCs

**Figure 1:**
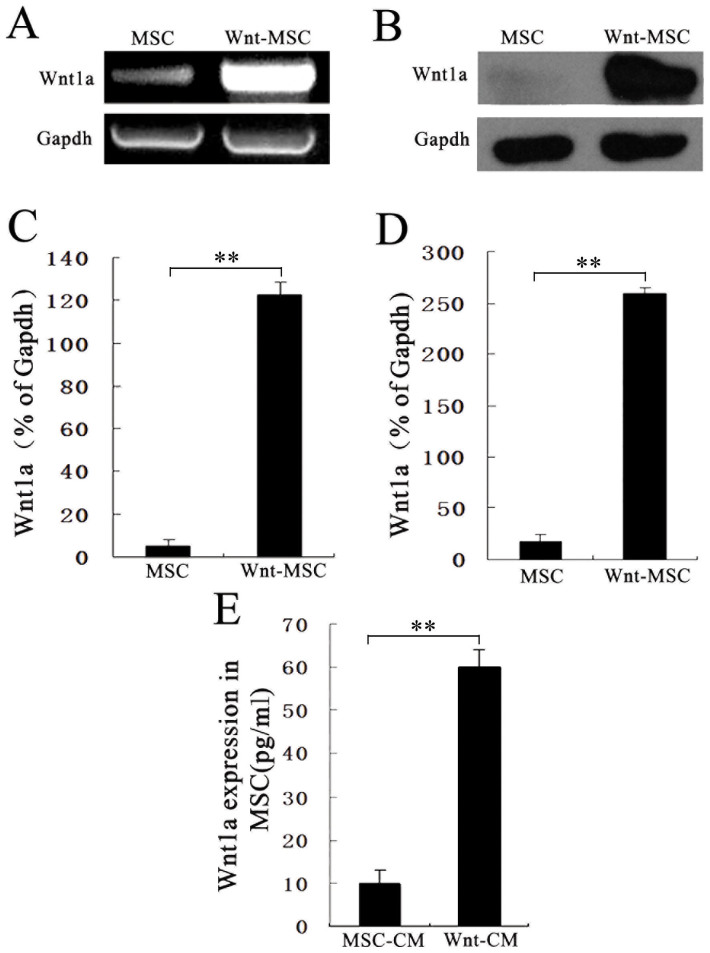
Wnt1a expression in BM-MSCs. (A), (C) RT-PCR analysis of Wnt1a expression and corresponding semi-quantitative analysis data. (B), (D) Wnt1a expression was validated by western blotting using anti-Wnt1a antibody and corresponding semi-quantitative analysis data. (E) Wnt1a expression levels of condition media (CM) from Wnt-MSCs and MSCs were measured by ELISA. The Wnt1a expression was up-regulated in Wnt-MSCs compared with MSCs. The data represents the means ± SEM, n = 3. *P < 0.05.

We generated a PWPT-Wnt1a retroviral expression system and propagated it in HEK293 cells. We purified and concentrated the retrovirus to use for increasing Wnt1a exposure in BM-MSCs with polybrene. We confirmed retroviral-mediated Wnt1a expression by qRT-PCR and western blotting. Wnt1a mRNA and protein levels were higher Wnt1a-treated BM-MSCs (Fig. 1A–D), whereas Wnt1a was weakly expressed in untreated control BM-MSCs. We also detected Wnt1a in culture supernatants by ELISA. Secreted Wnt1a protein levels were significantly higher in retroviral-Wnt1a-infected BM-MSCs compared to untreated control BM-MSCs (Fig. 1E). These data demonstrate that Wnt1a was successfully expressed in BM-MSCs and secreted into the medium of the cultured BM-MSCs.

### Wnt-CM promotes the hair follicle cycling

**Figure 2:**
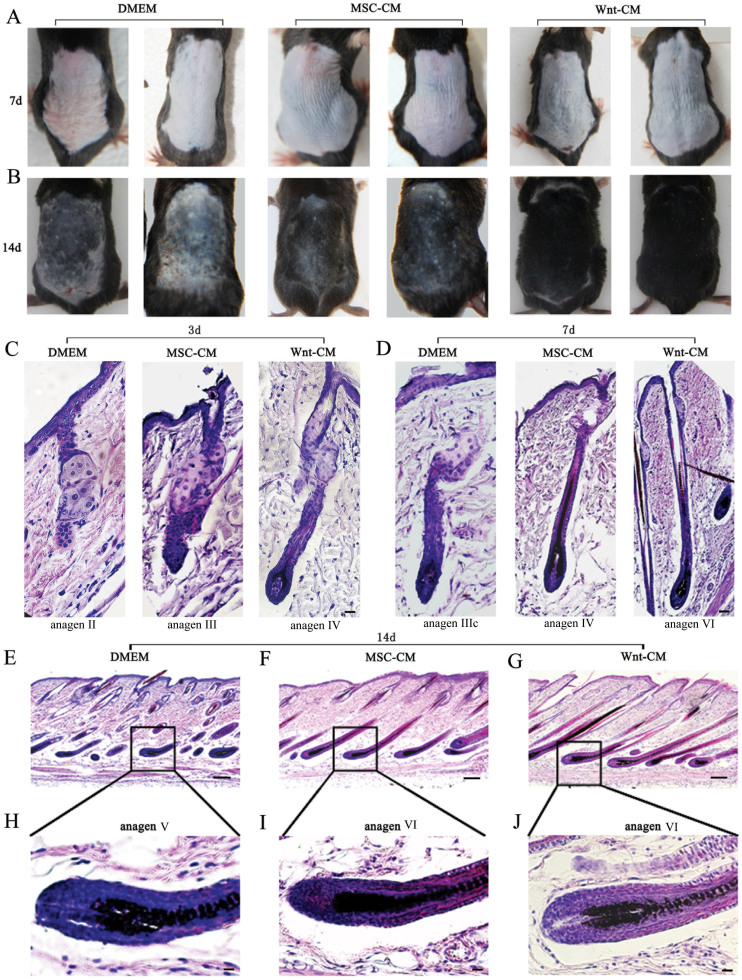
Wnt-CM promotes the hair follicle cycle in mice. Telogen C57BL/6 mice were intradermally injected with Wnt-CM. Typical photos of dorsal skin reveal that the stage of the hair cycle by observed comparison of dorsal skin colors and hair growth (A, B). HE staining indicates a structural change of hair follicles (C–J). (E), (F), (G) represent different stages in the hair cycles at 14 days by HE stained. (H), (I), (J) show a magnification of the framed labeled area. Scale bar (E–G) = 50 μm, (C), (D), (H–J) = 20 μm.

**Figure 3:**
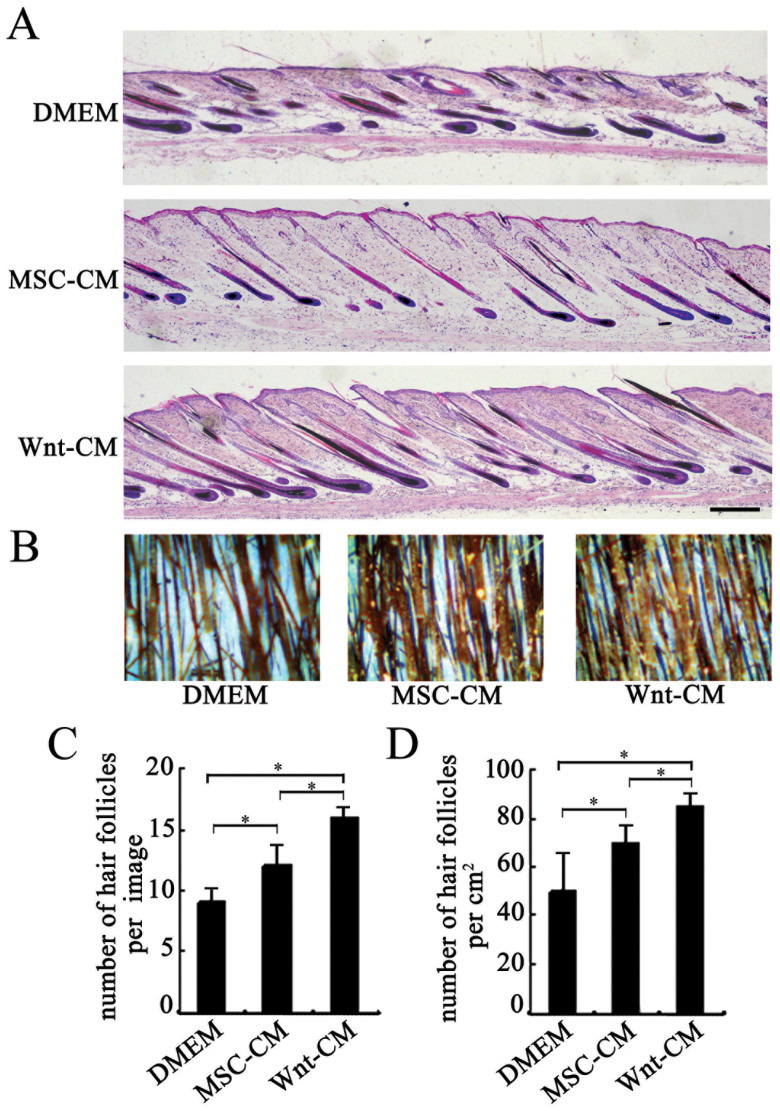
Wnt-CM increases the number of hairs in mice. (A) HE-stained dorsal skin sections at 14 days after depilation and (C) quantitative analysis of the number of hair follicles. (B) Typical photos of dorsal skin of C57BL/6 mice at 21 days after depilation and (D) quantitative analysis of the number of hair shafts. The data represent the means ± SEM, n = 3. *P < 0.05. Scale bar (A–C) = 100 μm.

To determine whether Wnt1a from BM-MSCs affects the hair follicle cycle, we collected the culture medium from Wnt1a-overexpressing BM-MSCs and prepared Wnt-CM by dialysis and concentrated it 20-fold. Then, 100 μl of Wnt-CM was injected through intradermally injected at multiple points in the dorsal skin in which there were mouse hairs that were clipped, with the same dose of MSC-CM and DMEM used as controls. We determined the hair cycle stage by measuring the amount by skin pigmentation. The results revealed that Wnt-CM treatment by 7 days diffuse darkening of the dorsal skin, and the control groups displayed no significant change (Fig. 2A). At 14 days, hair regrowth had completed in Wnt-CM-treated mice, and the tip of the hair shaft emerged through the epidermis while dorsal skin pigmentation was uneven and only contained a few hair shafts in the MSC-CM-treated group. The dorsal skin of the DMEM-treated group retained large areas without pigmentation (Fig. 2B). We harvested dorsal skin for histological analysis at 3, 7, and 14 days. The tissue was fixed in 4% formaldehyde to make paraffin sections (8 μm thick). Histomorphometrical analyses indicated that Wnt-CM promoted telogen-anagen transition (Fig. S1A). In particular, the hair follicles that were treated with Wnt-CM were transformed telogen to early anagen and middle-anagen at day 3 and day 7 (Fig. S1A). Remarkably, the Wnt-CM-treated group re-entered anagen prematurely compared with the DMEM-treated group an average 2–3 days. However, the progression of hair growth was not significantly changed after entering catagen (Figure S1B). HE staining revealed that the changing structural of different stages hair follicles in the Wnt-CM-treated group. Some of the hair follicles had entered anagen IV by day 3, hair follicles entered anagen V, and the hair shaft was completely developed by day 7. In addition, the regenerated hair erupted out of the epidermis and part of the hair follicle had entered anagen VI by day 14 (Fig. 3C–J). However, the MSC-CM-treated groups exhibited the hair follicles appeared to be in anagen III by day 3, the hair follicles entered anagen IV at day 7, and part of the hair follicle had entered anagen VI by day 14. The DMEM-treated group hair follicles entered anagen II, IIIc, andV at 3, 7 and 14 days, respectively. Overall, these results indicate that Wnt1a overexpression by BM-MSCs can induce hair to rapidly enter anagen of the hair cycle in mice.

### Wnt-CM increases the amount of hair regrowth

The number of hair was evaluated using typical photos of mouse dorsal skin and HE-stained skin tissue sections of mouse models subjected to Wnt-CM treatment and that of the mouse dorsal skin of control mice treated with MSC-CM and DMEM treated. The HE staining and local photos revealed the number of hair follicles and hair shafts, respectively (Fig. 3A and B). The number of hair follicles and hair shafts increased 1.5-fold (p < 0.05 n = 3) and 1.6-fold (p < 0.05 n = 3), respectively, in the Wnt-CM group compared with the DMEM group, and increased 1.2-fold and 1.3-fold, respectively, in the MSC-CM-treated group compared to the DMEM-treated group(Fig. 3C and D). Hair follicle cycling was augmented in the Wnt-CM-treated group compared to the MSC-CM and DMEM group. The results indicate that Wnt-CM increased the number of hair follicles by augmenting hair follicle cycling in the dorsal skin of mice.

### Wnt-CM triggers hair follicle regeneration

**Figure 4:**
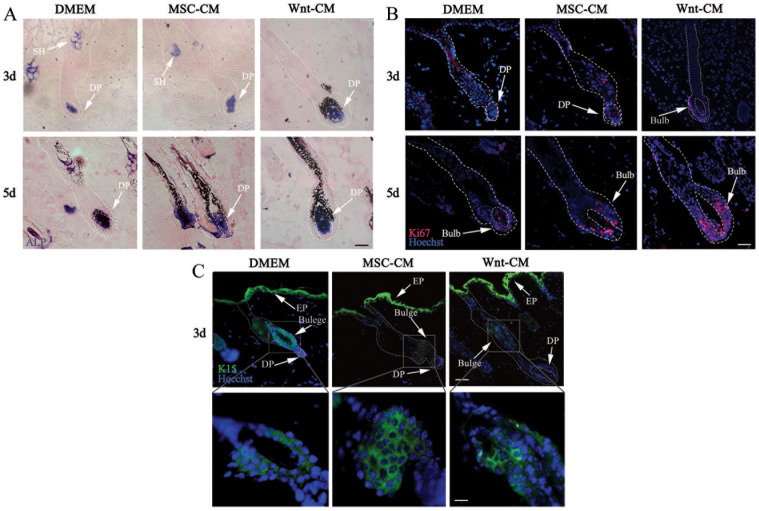
Expression patterns of structural markers in Wnt-CM-injected skin. (A) ALP activity was observed in the SG and DP at 3 and 5 days, Ki67 (B) at 3, 5days, and K15 (C) at 3 days. (D) shows a magnification of the framed labeled area. In each of the images, ALP (blue) is shown in blue, Hoechst (blue fluorescence), Ki67 (red fluorescence) and K15 (green fluorescence). The dashed line delineates the hair follicle structure. The arrowheads show the expression site of the DP (dermal papilla); SG, sebaceous gland; and EP, epidermis, scale bar (A–C) = 20 μm and (D) = 10 μm.

We clipped the dorsal hair during the first telogen phase to follow DP cell triggering of the hair stem cell proliferation in the hair follicle and subsequent hair regrowth. ALP, Ki67, and K15 are typically markers of anagen DP cells, cellular proliferation, and hair follicle stem cells, respectively, and they are expressed at different stages of hair regeneration. We analyzed the expression of ALP, Ki67, and K15 in the dorsal skin of Wnt-CM-treated, MSC-CM-treated and control mice using immunohistochemical analysis. Wnt-CM rapidly induced ALP expression in the SG (sebaceous gland) and DP area on day 3 and day 5 (Fig. 4A). With ALP enhanced expression, we observed enhanced Ki67 expression in the hair follicle bulb area of Wnt-CM-treated mice at day 3 and strong expression at day 5 (Fig. 4B). ALP expression was also enhanced in MSC-CM-treated mice at day 3, but there were fewer Ki67-positive cells compared to the Wnt-CM-treated group (Fig. 4B). K15 expression indicated that the hair was regrowing and the hair follicle stem cells were migration and differentiating in different-treated mice at day 3 (Fig. 4C). We did not observe any significant abnormalities in the epidermis, hair follicles, or other skin structures aside from ALP, Ki67 and K15 expression changes in all treated groups. These results indicate that Wnt1a from BM-MSCs can activate DP cells to accelerate hair follicle progression into the anagen, triggering hair regeneration.

### Wnt-CM upregulates the hair induction related genes *in vivo*

**Figure 5:**
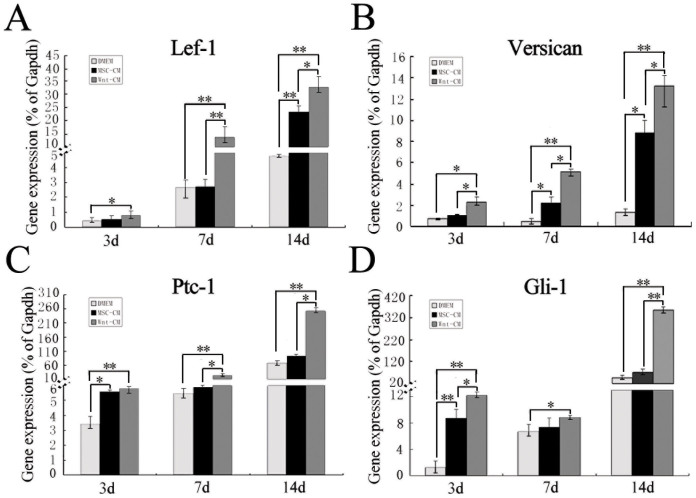
Hair follicle induction-related gene expression in the dorsal skin of mice. qRT-PCR analysis revealed the expression levels of genes of Lef-1 (A), Versican (B), Ptc-1 (C), and Gli-1 (D) in the dorsal skin of mice. The data represent the means ± SEM, n = 3. Statistically significant at *P < 0.05 and **P < 0.01.

To determine how Wnt-CM effects hair regeneration, we analyzed hair induction-related genes expression (Lef-1, Versican, Ptc-1 and Gli-1) by qRT-PCR of samples from the dorsal skin tissue of Wnt-CM-treated mice and MSC-CM-treated and DMEM-treated control mice. The results indicated that the expression of all hair induction-related genes was enhanced in the mice treated with Wnt-CM treated at day 3, and the expression rapidly increased by day 7. In addition, the increased expression tendency was sustained after 14 days. The MSC-CM-treated group also displayed increased expression compared to the DMEM group (Fig. 5A–D). These results suggest that Wnt1a from BM-MSCs could upregulate the expression of mice hair induction-related genes expression.

### Wnt-CM sustains hair induction related gene expression in cultured DP cells

**Figure 6:**
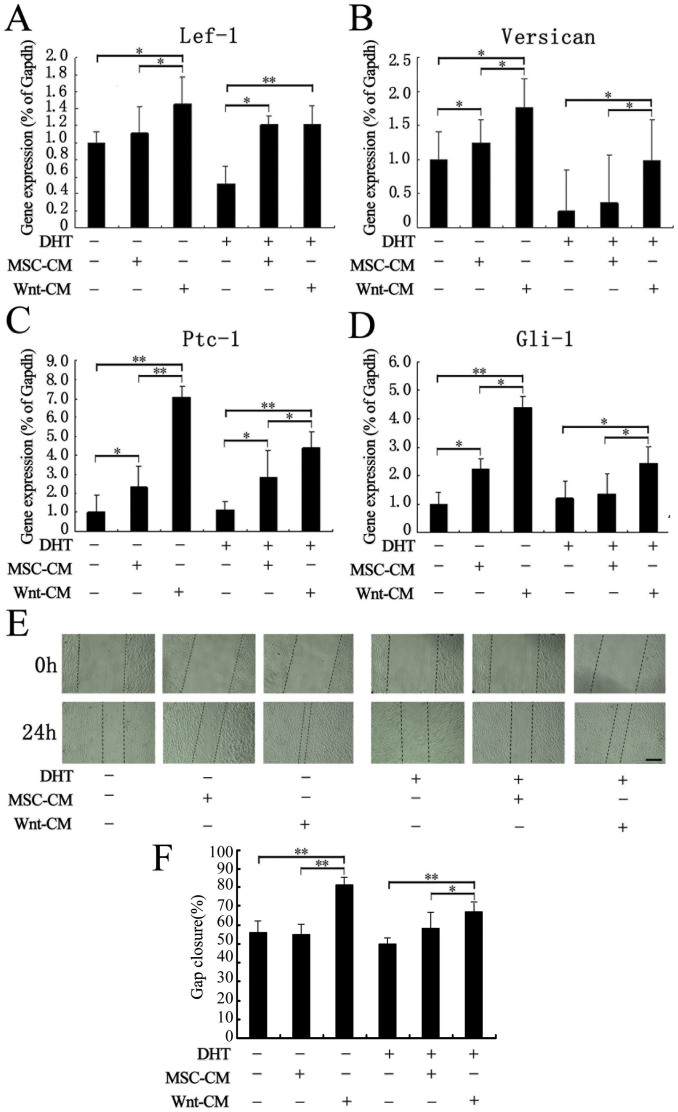
Wnt-CM enhances DP cell hair induction ability. (A–D) The qRT-PCR results revealed that Lef-1, Versican, Ptc-1, and Gli-1 were significantly downregulated in DHT-treated. THE expression of hair induction-related genes was enhanced by MSC-CM and Wnt-CM treatment. (E) The migration ability of DP cells was impaired by DHT according to the scratch test. (F) Quantitative analysis the scratch closure rate of DP cells cultured for 24 hours. The data represent the means ± SEM, n = 3. *P < 0.05 and **P < 0.01. Scale bar = 500 μm.

It is well known that microenvironment changes cause DP cells to lose their ability to induce hair follicle formation. Thus, we isolated, cultured and treated human scalp intermediate follicle DP cells with DHT to establish a model of DP cells with impaired hair induction ability. We examined Lef-1, Versican, Ptc-1 and Gli-1 expression by qRT-PCR. The results indicated that the hair induction-related gene expression of DP cells was significantly inhibited with 100 nM DHT treatment. We found that MSC-CM not only promoted hair growth gene expression but also recovered the hair hair induction-related gene expression of DP cells treated with DHT. Remarkably, Wnt-CM significantly increased hair induction-related gene expression ability was significantly enhanced by Wnt-CM treatment compared to MSC-CM treatment (Fig. 6A–D). We also investigated the effect of Wnt-CM on DP cells migration using scratch experiments (Fig. 6E). Our results revealed that Wnt-CM treatment significantly enhanced DP cell migration and restored the impaired migration ability of DP cells induced by DHT treatment. Although MSC-CM treatment also enhanced DP cell migration, its effect was weak compared to Wnt-CM treatment. These findings demonstrate that DP cells hair induction-related gene expression of can be maintained and repaired by MSCs *in vitro*, and this effect is significantly enhanced by Wnt1a overexpression in MSCs.

## Discussion

DP cells manage hair follicle cycling through secreted signaling factors. Human hair follicles affected by androgenic alopecia contain a largely intact population of hair follicle stem cells and a primary defect in DP signaling, resulting in hair anagen momentum being unable to start. The restoration of DP cell hair induction ability has been considered a potential therapy for hair loss^20^. In this study, we demonstrated that Wnt1a overexpression by BM-MSCs could induce the ransition of hair follicles from telogen to anagen significantly increases the number of mice hairs and restores the expression of hair induction-related genes damaged by DHT *in vitro*. These finding indicate that Wnt1a overexpression by BM-MSCs can induce hair follicle regeneration.

Hair cycling between telogen to anagen is precisely regulated by DP cells. The Wnts pathway is an important signaling pathway that activates the hair induction ability of DP cells^21^. Wnt7a can mediate epidermal-mesenchymal interactions that enhance wound-induced follicle neogenesis in mouse models, and it is also important in maintaining the hair induction activity of cultured DP cells^10^,^11^. Adenovirus-mediated ectopic expression of other Wnt proteins, such as Wnt10b, can activate precocious anagen entry in mouse models^12^. During chicken embryo feather formation, Wnt1 is first expressed in the epidermis and then becomes enhanced on the embryonic 6th day during dermis formation, and the primary feather row appears on the embryonic 7.5th day along the dorsal midline of the lumbar region. There is complete loss of Wnt1 complete loss of expression by the embryonic 8.5th day. During feather formation, Wnt1 triggers the formation of a feather and induces the appendage dermis^13^,^22^. Therefore, Wnt1a expression is perhaps the original signal in hair formation to regulate the hair cycle and regeneration. In our results, Wnt-CM could enhance the expression of hair induction-related proteins and genes. Lef-1 is an essential regulatory protein in the Wnt signaling pathway that controls cell growth and differentiation^1^. Wnt-CM can up-regulate Lef-1expression in mouse model dorsal skin tissue depilation and in cultured DP cells treated with Wnt-CM. The data demonstrated that Wnt1a from BM-MSCs activates the Wnt/β-catenin signaling pathway to mediate hair regeneration.

The hair cycle and regeneration were complex process of cyclic tissue remodeling that involves growth factors, cytokines, hormones, adhesion molecules and related enzymes^23^. In previous studies, vascular endothelial growth factorc (VEGF) incorporated into collagen hydrogel could accelerate hair follicle entry into anagen phase and enhance hair elongation when it was subcutaneously implanted into the back of mice. Thede results demonstrated that VEGF can promote hair follicle growth through induced angiogenesis^24^. Insulin-like growth factor-1 (IGF-1) is expressed in the hair follicle and was demonstrated to affect follicular proliferation, tissue remodeling, and the hair growth cycle. Loss of IGF-1 induced a delay telogen and retarded the onset of the second anagen phase, and the guard hairs of transgenic mice skin significantly elongated. However, the ectopic expression of IGF-1 in transgenic mice skin resulted in partial compensation of the hair growth cycle^25^. Platelet-derived growth factor (PDGF) has been demonstrated to induce entry into the anagen phase of the hair growth cycle at the injection sites of the dorsal skin of C3H mice through its receptors in the hair follicle epithelium. When the PDGF gene was knocked out, the mice exhibited a thinner dermis, misshaped hair follicles, smaller dermal papillae, abnormal dermal sheaths and thinner hair compared to wild-type siblings^26^. MSCs are multipotent cells that have been widely applied in regenerative medicine. *In vitro and in vivo* studies have increasingly shown that MSCs secretions have an increasingly wide range of biological functions. MSCs secrete many factors useful in regenerative and repair processes, which creates a comfortable environment for the cells in adjacent tissues^27^,^28^. In this study, we concentrated the culture supernatant of MSCs and prepared conditioned medium (MSC-CM). MSC-CM accelerated the hair cycle and increased hair induction-related gene expression *in vivo* and restored the hair induction ability of DP cells that had been damaged by *in vitro*. Although we did not identify the individual roles of individual factors, our results suggest that BM-MSCs release a large number of growth factors that may be beneficial toward activating the hair induction ability of dermal papilla cells to support hair follicles. MSCs genetically modified to secrete certain biological factors have been reported to stimulate the cartilage formation in animal models. Alterations in Wnt signaling may maintain the progenitor pool and the regulate of differentiation and lineage commitment. Therefore, selective change in the Wnt signal pathway may be a useful a target for cell-based therapy. The combined regulation seceral genes may help us determine the method of hair regeneration. Once these problems are solved, the coordinated expression of multiple genes in MSCs using complex regulatory systems could fulfilling the potential of gene therapy for stem-cell-based hair regeneration Wnt1a and MSCs treatment synergy produces better results than MSC-CM treatment alone.

The DP plays key roles in epithelial-mesenchymal interactions to enable hair follicle development, regeneration, and specification of hair size, shape and cycling^29^. Previous studies have demonstrated that an altere DP cell microenvironment can lead to hair loss in human skin, such as androgenic hair loss and chemically induced hair loss^20^,^30^. In addition, rat DP cells cultured in DMEM containing 10% FBS *in vitro* have been demonstrated to retain their original hair growth induction ability until passage 3, and the loss is due to the unfavorable DP cell environmental change^30^. To preserve the properties of DP cells, many strategies aim to mimic the *in* *vivo* microenvironment, such as culturing DP cells in three-dimensional aggregates or culturing them together with keratinocytes on extracellular matrix substrates^31^. Furthermore, the hair induction ability of DP cells can be maintained by co-culturing with Wnt3a-expressing feeder cells. These results demonstrate that Wnt3a signaling has favorable effects on the follicle induction ability of DP cells^10^. In our study, we found that Wnt-CM prepared by Wnt1a overexpression by BM-MSCs can enhance the expression of hair induction-related genes of DP cells in vitro. In addition, we found that hair induction-related genes displayed a downward expression trend at in DP cells treated with DHT, and this declining trend can be reversed with Wnt-CM treatment. These observations prove that Wnt1a and MSCs are suitable for mimicking the microenvironment necessary for restoration and enhancement of DP cell hair induction ability. Our results also provide a theoretical foundation for culturing a large number of DP cells for potential alopecia therapies.

In summary, Wnt-CM developed from Wnt1a overexpression in MSCs can accelerate the initiation of the hair follicle transition from the telogen phase to the anagen phase, increase the number of hairs and enhance expression of hair induction-related proteins *in vivo*. Furthermore, Wnt-CM can restore and promote the hair induction ability of DP cells that has been impaired by DHT *in vitro*. Our findings indicate that Wnt1a from MSCs can restore the ability of DP cells to induce hair follicle regeneration and can potentially serve as the basis of alternative therapeutic methods for alopecia.

## Methods

### Animals

Six-week-old male (SD) rats (220–250 g) and six-week-old male C57BL/6 mice were obtained from the Chinese PLA General Hospital. The protocol was approved by the medical ethics committee of the Chinese PLA General Hospital.

### Amplification and purification of retroviral vectors

Murine Wnt1a cDNA (NM_021279, oriGene, MD, USA) was inserted into the Mlu I and Sal I sites of a retrovirus vector (PWPT, gift from Xin Wang, Shanghai Institute of Biochemistry and Cell Biology). Recombinant vectors were amplified in HEK293FT cells to generate high-titer preparations and purified using polyethylene glycol 6000 (PEG) via the precipitation method^32^. Wnt1a virus was dissolved in PBS (phosphate-buffered saline) buffer and stored at −80°C.

### Rat BM-MSCs isolation and Wnt-CM preparation

BM-MSCs were isolated and harvested from the femurs of 6-week-old male (SD) rats (220–250 g), purified, and identified as described previously^33^. The MSCs were cultured to 50%–60% confluence in T75 culture flasks and treated with the Wnt1a virus-containing medium combined with Polybrene (8 mg/mL), which was applied to the container, which was replenished with 12 mL of serum-free DMEM (GIBCO, NY, USA) for 24 h prior to harvesting the media of the Wnt1a virus-containing BM-MSCs and BM-MSCs. The collected media samples were centrifuged at 3,000 rpm for 10 min to remove the cell debris. The media were concentrated 20-fold by dialysis with a 7-kDa molecular weight cut-off bag filter (Union Carbide, CT, USA) in PEG 20000 at 4°C to prepare the Wnt-CM and MSC-CM.

### ELISA

The level of Wnt1a protein in cell culture supernatants from MSCs was assayed using an Wnt1a immunoassay kit (CUSABIO, WH, CN). After transfection, conditioned medium was collected. MSC-CM or Wnt-CM was added to each well and incubated for 2 h at room temperature. The Wnt1a expression level was determined according to the manufacturer's protocol, and absorbance was measured at 450 nm using an ELISA reader (BIOTEK, VT, USA).

### DP cell isolation, culture and DHT induction

Intermediate follicles^34^, appeared thinner and less pigmented than terminal follicles and were obtained from 3 mm punch biopsies taken from the balding scalp of male patients undergoing hair transplantation surgery. Ethical permission and informed consent were obtained preoperatively. DP cells were isolated from hair scalp follicles according to previously described method^35^. The cells were maintained in 10 ml of DMEM (Gibco, NY, USA) supplemented with 10% fetal bovine serum (Hyclone, UT, USA). On passage 2, the DP cells were grown to 50%–60% on 24-well plates containing 100 nM DHT DMEM medium and induced for 24 h. After which, 20-fold conditioned medium (100 μl per 1 ml of DMEM) was added, and the cells were cultured for another 24 h. The DP cells were harvested, and the relevant gene expression levels were assayed^7^.

### Hair regeneration model

Six-week-old male C57BL/6 mice were obtained and then allowed to adapt for a week with food and water. Anagen was induced by depilation of skin on the back of C57BL/6 mice that were in the telogen phase of the cycle, as described^36^. At one day after hair removal, 100 μl of Wnt-CM, MSC-CM, DMEM (Gibco BRL, NY, USA) was intradermally injected into multiple points.

### ALP (alkaline phosphatase) activity

The detection of alkaline phosphatase (ALP) activity in frozen sections (8 μm thick) was performed with an ALP detection kit (Chemicon Millipore, Temecula, CA, USA), following the manufacturer's protocol. Briefly, frozen sections were fixed with 4% paraformaldehyde, rinsed with phosphate-buffered saline (PBS), and incubated with a solution containing Fast Red Violet and Naphthol AS-BI phosphate for 15 min in the dark at room temperature. The reaction was terminated by rinsing again in phosphate-buffered saline (PBS). The sections were mounted in Permount (eBioscience, CA, USA).

### Quantitative real-time RT-PCR (qRT-PCR)

The treated skin tissue and DP cell total RNA were isolated using an RNeasy Mini Kit (Qiagen, CA, USA). Single-stranded cDNA was synthesized using SuperScript II reverse-transcriptase and oligo (dT) (Invitrogen, CA, USA). The qRT-PCR was used to determine transcript expression and normalized against the glyceraldehyde-3-phosphate dehydrogenase (Gapdh). The primer sets used are shown in Table 1.

### Western blot analysis

Western blotting was performed as previously described^37^. MSCs and retrovirally infected MSC total protein was obtained. The primary antibodies were used of rabbit polyclonal antibodies to Wnt-1 and mouse monoclonal antibodies to Gapdh (Abcam, MA, UK), and the used secondary antibodies used goat anti-rabbit and goat anti-mouse IgG (HRP). The blots were analyzed with densitometry using ImageJ software (NIH, MD, USA).

### Histological analysis

Dorsal skins was harvested for histological analysis. The treated dorsal skins were fixed in 4% formaldehyde and then processed by paraffin block embedding using standard techniques to make Paraffin sections (8 μm thick). The general histology was visualized by hematoxylin-eosin (HE) staining and was observed using a microscopy (Olympus BX53JP).

### Immunohistochemistry

Dorsal skins were harvested, fixed in 4% paraformaldehyde, dehydrated with sucrose and embedded in OCT. The frozen sections (8 μm thick) were incubated with rabbit polyclonal anti-mouse K15 (Abcam, MA, UK), rabbit polyclonal anti-mouse Ki67 (Abcam, MA, UK), (1:200) for 4°C overnight. The sections were then washed in PBS with Tween 20 and incubated with Alexa Fluor 488-conjugated goat anti-rabbit IgG and Alexa Fluor 594-conjugated goat anti-rabbit IgG (Invitrogen, CA, USA), respectively, for 1 h. Subsequently, frozen sections were stained with Hoechst (Vector, Burlingame, CA) at a 1:2000 level. The sections were observed via fluorescence microscopy (Olympus BX53, JP).

### Quantitative histomorphometry

Hair cycle stages were evaluated and classified as described by the means of quantitative histomorphometry^36^. Histomorphometry was performed with HE-stained sections that were taken from defined back skin regions. At least 50 hair follicles per mouse per group were evaluated^38^.

### Cell scratch experiment

DP cells were plated in 6-well plates at 5 × 105 cells/well. When cells covered the bottom of the well, a scratch was made with a sterilized P1000 pipette tip (a width of 1–1.2 mm), and the DP cells continued to be cultured in the different media after washing with PBS. After 24 h, the scratches were analyzed via microscopy (Olympus BX53, JP), and the scratch area was measured using IPP software (Media Cybernetics).

### Statistics

All data from the quantitative experiments are presented as the mean plus standard deviation (SD). All experiments were repeated three times with independent cultures, and similar results were obtained. Statistical significance was determined using Student's t-test. Statistical significance was accepted as P < 0.05.

## Author Contributions

H.H., X.F., W.H. designed the research. L.D. and L.X., H.H. and J.L. performed the experiments. D.T., C.T., Q.H., Q.H. and L.D., date analysis, Y.Z., H.L. prepared figures 1–6. L.D. and L.X., H.H. contributed equally in writing the manuscript. All of the authors reviewed and approved the manuscript.

## Supplementary Material

Supplementary Information Supplementary Figure 1

## Figures and Tables

**Table 1:** List of sequences of forward and reverse primers

Genes	Primer	Product	GeneBank
	Sequences	size (bp)	Accession No.
Lef-1	Forward: 5′-> 3′gccaccgatgagatgatccc	107	NM_010703
	Reverse: 5′-> 3′ttgatgtcggctaagtcgcc		
	Forward: 5′-> 3tgccaaatatgaataacgaccca	150	NM_001166119
	Reverse: 5′-> 3gagaaaagtgctcgtcactgt		
Versican	Forward: 5′-> 3′ttttacccgagttaccagactca	106	NM_001081249
	Reverse: 5′-> 3′ggagtagttgttacatccgttgc		
	Forward: 5′-> 3′gtaacccatgcgctacataaagt	110	NM_001126336
	Reverse: 5′-> 3′ggcaaagtaggcatcgttgaaa		
Ptc-1	Forward: 5′-> 3′aaagaactgcggcaagtttttg	164	NM_008957
	Reverse: 5′->3′cttctcctatcttctgacgggt		
	Forward: 5′-> 3′ccagaaagtatatgcactggca	134	NM_001083603
	Reverse: 5′->3′gtgctcgtacatttgcttggg		
Gli-1	Forward: 5′-> 3′ccaagccaactttatgtcaggg	130	NM_010296
	Reverse: 5′-> 3′agcccgcttctttgttaatttga		
	Forward: 5′-> 3′agcgtgagcctgaatctgtg	188	NM_001167609
	Reverse: 5′->3′cagcatgtactgggctttgaa		
Wnt1a	Forward: 5′-> 3′ggtttctactacgttgctactgg	121	NM_021279
	Reverse: 5′-> 3′ggaatccgtcaacaggttcgt		
Gapdh	Forward: 5′-> 3′aggtcggtgtgaacggatttg	123	NM_008084
	Reverse: 5′-> 3′tgtagaccatgtagttgaggtc		
	Forward: 5′-> 3′acaactttggtatcgtggaagg	101	NM_001256799
	Reverse: 5′->3′gccatcacgccacagtttc		

PCR amplification conditions on the Applied Biosystems 7500 Real Time PCR System: 58°C for 5 minutes; 95°C for 2 minutes; 40 cycles of 95°C for 15 seconds and 60°C for 60 seconds.
