# Supplementary material for: Treatment of MSCs with Wnt1a-conditioned medium activates DP cells and promotes hair follicle regrowth
Source: Sci Rep. 2014 Jun 25;4:5432. doi: 10.1038/srep05432 (PMC4069670; doi:10.1038/srep05432)
Supplement: Supplementary Information — Supplementary Figure 1 [file srep05432-s1.pdf]

Treatment of MSCs with Wnt1a-conditioned medium activates DP cells and promotes hair follicle regrowth

Liang Dong[a,d], Haojie Hao[a,d], Lei Xia[b,d]， Jiejie Liu[a], Dongdong Ti[a], Chuan Tong[a], Qian Hou[a], Qingwang Han[a], Yali Zhao[c], Huiling Liu[a]， Xiaobing Fu[a*], and Weidong Han[a*]

[a]*Institute of Basic Medicine Science, College of Life Science, Chinese PLA General Hospital, Beijing 100853, China;* [b]*Department of Medical Administration，Chinese PLA General Hospital, Beijing 100853, China;* [c]*Central laboratory, Hainan branch of Chinese PLA General Hospital, Sanya, 572013, China*

[d]*These authors contributed equally to this work*

*Corresponding author. Tel.: +86-10-66937463 Fax: +86-10-66937516:E-mail: hanwdrsw69@yahoo.com (Weidong Han). Tel.: +86-10-66939569 fax: +86-10-68216489 E-mail: fuxiaobing@vip.sina.com (Xiaobing Fu).

A

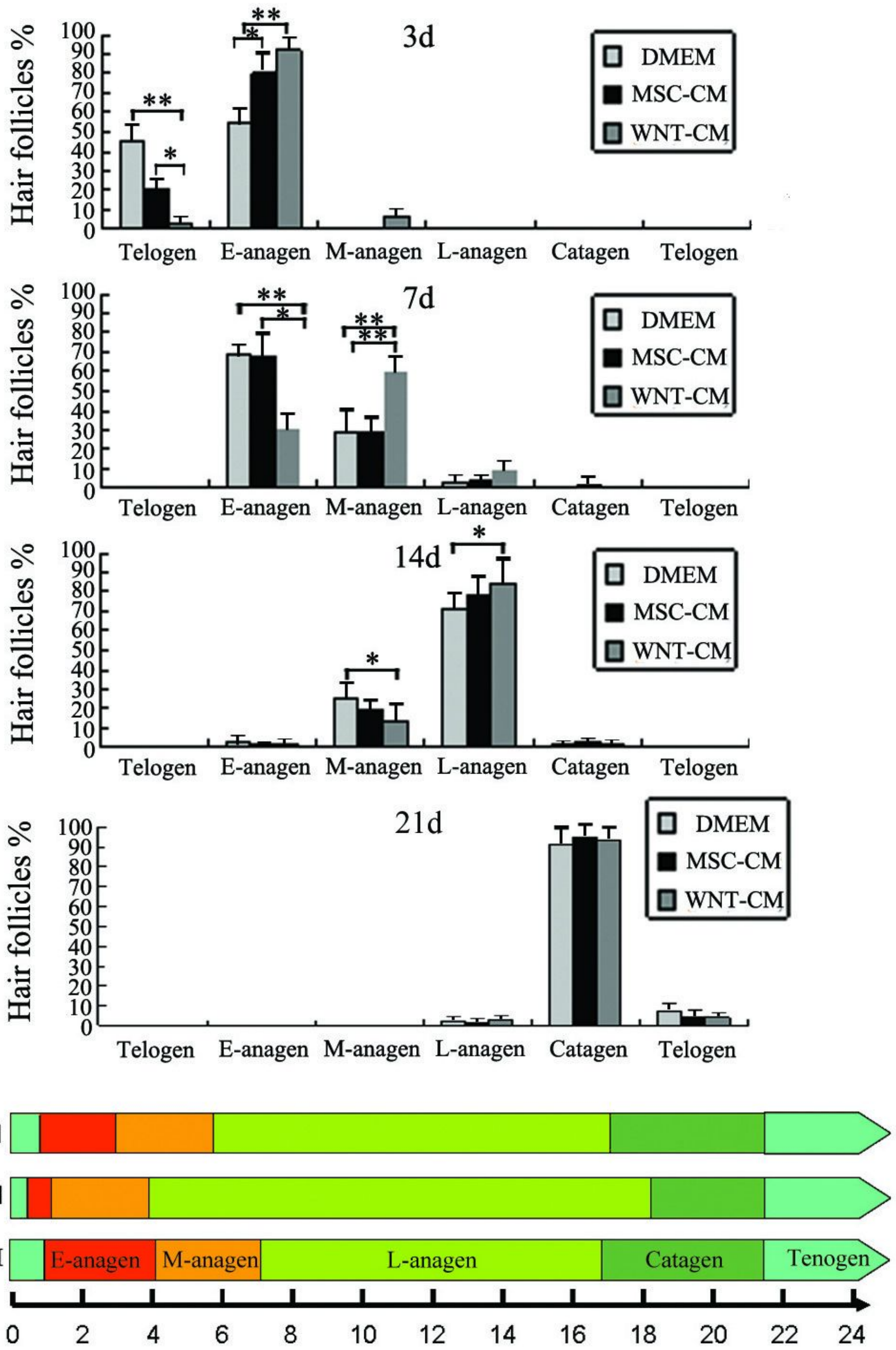

Supplementary Figure 1. Effects of Wnt-CM on hair re-growth and hair cycle stage in mouse skin. (A) Quantitative histomorphometric analyses that the numbers of the hair follicles at different stages in skin treated with Wnt-Cm, MSC-CM and DMEM for 3, 7, 14, and 21 d were performed with three mice for each group (n=3

mice per group; Early anagen includes anagen I-II; Middle anagen includes anagen III-IV; Late anagen includes anagen V-anagen VI; catagen; tenogen). (B) Schematic illustrating hair follicle cycle in Wnt-CM, MSC-CM and DMEM treatment group mice 0-24 d. The data represent the means ± SEM, n = 3. *P <0.05 and **P <0.01.
